# IFN-γ and IgG responses to *Mycobacterium tuberculosis* latency antigen Rv2626c differentiate remote from recent tuberculosis infection

**DOI:** 10.1038/s41598-020-64428-z

**Published:** 2020-05-04

**Authors:** Nicolás O. Amiano, María P. Morelli, Joaquín M. Pellegrini, Nancy L. Tateosian, Agustín Rolandelli, Vanesa Seery, Florencia A. Castello, Claudio Gallego, Rita Armitano, Juan Stupka, María A. Erschen, Lorena M. Ciallella, Graciela C. de Casado, Liliana Cusmano, Domingo J. Palmero, Juan L. Iovanna, Verónica E. García

**Affiliations:** 1 Instituto de Química Biológica, Facultad de Ciencias Exactas y Naturales (IQUIBICEN), Universidad de Buenos Aires (UBA)-CONICET, Intendente Güiraldes 2160, Pabellón II, 4° piso, Ciudad Universitaria (C1428EGA), Buenos Aires, Argentina; 2 Departamento de Química Biológica, Facultad de Ciencias Exactas y Naturales, UBA, Intendente Güiraldes 2160, Pabellón II, 4° piso, Ciudad Universitaria (C1428EGA), Buenos Aires, Argentina; 3 Hospital General de Agudos Parmenio Piñero, Av. Varela 1301, (C1406EKZ) Buenos Aires, Argentina; 4 División Tisioneumonología Hospital F.J. Muñiz, Uspallata 2272, (C1282AEN) Buenos Aires, Argentina; 5 Centre de Recherche en Cancérologie de Marseille (CRCM), INSERM U1068, CNRS UMR 7258, Aix-Marseille Université and Institut Paoli-Calmettes, Parc Scientifique et Technologique de Luminy, Marseille, France

**Keywords:** Biological techniques, Biotechnology, Immunology, Biomarkers, Diseases

## Abstract

Tuberculin skin test (TST) and IFN-γ release assays are currently used to detect *Mycobacterium tuberculosis* (*Mtb*) infection but none of them differentiate active from latent infection (LTBI). Since improved tests to diagnose *Mtb* infection are required, we studied the immune response to *Mtb* latency antigen Rv2626c in individuals exposed to the bacteria during different periods. Tuberculosis patients (TB), TB close contacts (CC: subjects exposed to *Mtb* for less than three months) and healthcare workers (HW: individuals exposed to *Mtb* at least two years) were recruited and QuantiFERON (QFT) assay, TST and IFN-γ secretion to Rv2626c were analyzed. Twenty-two percent of the individuals assessed had discordant results between QFT and TST tests. Furthermore, QFT negative and QFT positive individuals produced differential levels of IFN-γ against Rv2626c, in direct association with their exposure period to *Mtb*. Actually, 91% of CC QFT negative subjects secreted low levels of IFN-γ to Rv2626c, whereas 43% of HW QFT negative people produced elevated IFN-γ amounts against Rv2626c. Conversely, 69% of CC QFT positive subjects didn´t produce IFN-γ to Rv2626c. Interestingly, a similar pattern of IgG anti-Rv2626c plasma levels was observed. Therefore, determination of IFN-γ and IgG levels against the dormancy antigen Rv2626c allows to identify established LTBI.

## Introduction

Tuberculosis (TB) is the leading cause of decease by a single infectious agent and one of the top 10 causes of death worldwide. In 2018, the World Health Organization estimated 10 million new cases and 1.45 million deaths^1^. Furthermore, 1.7 billion people are considered latently infected with *Mycobacterium tuberculosis* (*Mtb)*^1^, representing a vast reservoir of the bacteria. Thus, there is an urgent need for developing rapid methods to detect *Mtb* infection. Tuberculin skin test (TST) is a commonly used screening method for *Mtb* infection^2^. However, TST displays a limited specificity and cannot distinguish active from latent TB infection^3^. Additionally, TB blood tests or interferon-gamma release assays (IGRAs) are other type of methods for detection of *Mtb* infection. Current IGRAs employ ESAT-6 and CFP-10, two antigens encoded in the region of difference 1 locus present in both *Mtb* and in *M. bovis* genomes^4^. Importantly, these antigens are missing in Bacillus Calmette-Guerin (BCG) vaccine and most environmental mycobacteria, which makes IGRAs more specific than TST^4^. However, both assays only detect individuals who has been infected with *Mtb*, but they do not differentiate between latent and active *Mtb* infection^5,6^. Therefore, the use of other distinctive *Mtb* antigens in blood tests is urgently required^7^.

During dormancy, the bacilli are contained in granulomas due to the active role of the immune system and the deprivation of nutrients and oxygen^8^. Hypoxia adaptation of the bacteria includes the expression of different proteins encoded in the DosR regulon^9^, which enables *Mtb* to successfully shift between aerobic and non-aerobic conditions^10^. Although the exact functions of numerous latency proteins still remain unknown, several of them have been analyzed as potential diagnostic markers^11^. Previously, we reported that the immune response to the *Mtb* dormancy antigen Rv2626c, differentiates latently infected BCG-vaccinated individuals from TB patients and healthy donors. Our findings demonstrated that specific epitopes from Rv2626c induced the secretion of significant amounts of IFN-γ in latently infected individuals (LTBI) in sharp contrast to non-infected individuals^12^. In the present work, we investigated the effect of the exposure time to *Mtb* on the human immune response against Rv2626c antigen. Furthermore, besides the analysis of IFN-γ production against Rv2626c, we investigated the specific IgG plasma levels to this antigen.

Altogether, our results demonstrate that both IFN-γ and IgG responses against Rv2626c allow discriminating subjects with established latent *Mtb* infection from individuals recently exposed to the pathogen. These findings might represent an advantageous tool for the improvement of established LTBI diagnosis.

## Results

Eighty-three close contacts (CC) of TB patients, 114 healthcare workers (HW) highly exposed to *Mtb* and 42 subjects with active TB were studied. Age, sex, TST and QuantiFERON-TB Gold Plus kit (QFT) results are shown in Table 1. Briefly, CC comprised subjects who had lived or worked with recently diagnosed pulmonary TB patients for less than three months during 6 or more hours each day; HW included physicians and nurses who had worked at Hospital areas where TB patients were confined at least for two years; TB patients were subjects diagnosed with active disease (90% of them displayed acid-fast bacilli (AFB) smear-positive sputum).

**Table 1:** Population characteristics.

Study Subjects Characteristic		Group 1: Close Contacts (CC) (N = 83)	Group 2: Healthcare Workers (HW) (N = 114)	Group 3: Tuberculosis Patients (TB) (N = 42)
Age (years), median (range)		38 (18–77)	39 (23–64)	30 (18–74)
Sex	Male	35 (42)	50 (44)	33 (79)
	Female	48 (58)	64 (56)	9 (21)
QFT	(+)	33 (40)	38 (33)	34 (81)
	(−)	46 (55)	76 (67)	5 (12)
	Indeterminate	4 (5)	0 (0)	3 (7)
TST	(+)	17 (25)	17 (22)	18 (75)
	(−)	50 (75)	60 (78)	6 (25)
AFB in sputum smear	(+)	--------	--------	38 (90)
	(−)	--------	--------	4 (10)

Note: Values were expressed as number (percentage). AFB: Acid-Fast Baclli.

### Comparison of QuantiFERON-TB gold plus kit and TST results

**Figure 1:**
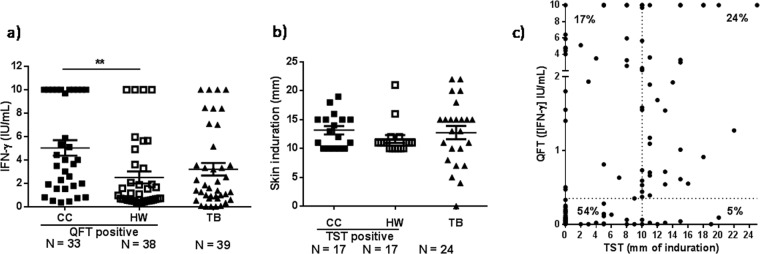
QFT and TST analyses. (a) IFN-γ levels as measured by QFT. The levels of IFN-γ were assayed in whole blood from CC and HW QFT positive individuals and TB patients participating in the study by using the QFT kit. IFN-γ levels are expressed as IU/mL (IU_**IFN-γ**_/mL = IU_**IFN-γ**_/mL detected in TB1 or TB2 antigen tube – IU_**IFN-γ**_/mL measured in Nil tube). (**b)** Diameter of induration as measured by the Tuberculin Skin Test (TST). Purified protein derivative (PPD) was administered and 48–72 h after the injection, the skin test reaction was measured as mm of the induration. Mean ± SEM for each group of subjects is shown. **P < 0.01. *P*-values were calculated by Kruskal-Wallis test and Dunn’s multiple comparisons post-test for unpaired samples. Each symbol represents an individual. (**c)** Scatter plot of IFN-γ levels (as measured by QFT) and diameter of induration (as measured by TST). IFN-γ levels were assayed in whole blood using the QFT kit and were expressed as IU/mL. The skin test reaction was measured as mm of the induration after 48–72 h of PPD injection. The vertical dotted line represents the TST cut-off (10 mm); the horizontal dotted line indicates the QFT cut-off (0.35 IU_IFN-γ_/mL). Percent Concordant = 78%; Percent Discordant = 22%. *Kappa* = 0.530. SE of *kappa* = 0.067. Moderate agreement.

We initially analyzed the levels of IFN-γ produced by CC, HW and TB patients by using the QuantiFERON-TB Gold Plus kit (QFT). In addition, we recorded the skin induration after TST administration by trained personnel. Our results showed that CC QFT positive individuals secreted significant higher levels of IFN-γ than HW QFT positive subjects (Fig. 1a). In contrast, no differences in the skin induration were detected between CC TST positive and HW TST positive groups (Fig. 1b). These results suggest that the IFN-γ response induced against QFT antigens (ESAT-6 and CFP-10) fluctuates according to the exposure time of subject to *Mtb*, whereas the delayed hypersensitivity to tuberculin is independent of the exposure time of the individual to the pathogen.

Next, we analyzed the correlation between the QFT and TST results from 167 out of 239 individuals that participated in the study. We observed that 54% of the subjects displayed negative results for both tests (double negative individuals) (Skin induration < 10 mm and [IFN-γ]_QFT_ < 0.35 IU/mL) whereas 24% of the individuals presented positive results for both assays (double positive persons) (Fig. 1c). In addition, we also detected an important discrepancy between the two assays: 17% of the individuals displayed negative TST but positive QFT, whereas 5% of the subjects presented negative results for QFT but positive TST (Percent  Discordant = 22%). The analysis of multivariate categorical data using Cohen’s kappa coefficient (κ) showed 78% of coincident results for both tests (*kappa* = 0.530; Standard Error (SE) of kappa = 0.067). Considering that κ < 0.60 indicates a moderate agreement among the raters^12^, the discordant findings between QFT and TST suggest that the diagnosis of LTBI (traditionally determined by QFT positive and / or TST positive results in the absence of TB disease) needs to be improved.

**Figure 2:**
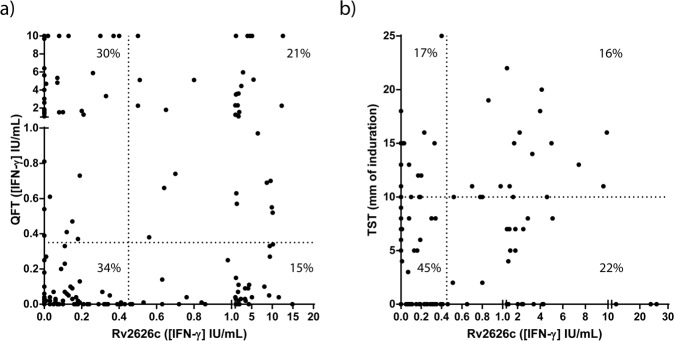
(**a**) Scatter plot of IFN-γ levels as measured by QFT and by Rv2626c IGRA. IFN-γ levels in whole blood of participants were measured by using the QFT kit and the Rv2626c IGRA assay. The vertical dotted line represents the Rv2626c IGRA cut-off (0.45 IU_IFN-γ_/mL); the horizontal dotted line indicates the QFT cut-off (0.35 IU_IFN-γ_/mL). Percent Concordant = 55%; Percent Discordant = 45%. *Kappa* = 0.106. SE of kappa = 0.066. Slight agreement. (**b)** Scatter plot of mm of induration measured by TST and IFN-γ levels measured by the Rv2626c IGRA. IFN-γ levels in whole blood of participants were measured by using the Rv2626c IGRA assay. The vertical dotted line represents the Rv2626c IGRA cut-off (0.45 IU_IFN-γ_/mL). The skin test reaction was measured as mm of the induration after 48–72 h of PPD injection. The vertical dotted line represents the TST cut-off (10 mm). Percent Concordant = 61%; Percent Discordant = 39%. *Kappa* = 0.152. SE of *kappa* = 0.092. Slight agreement.

**Figure 3:**
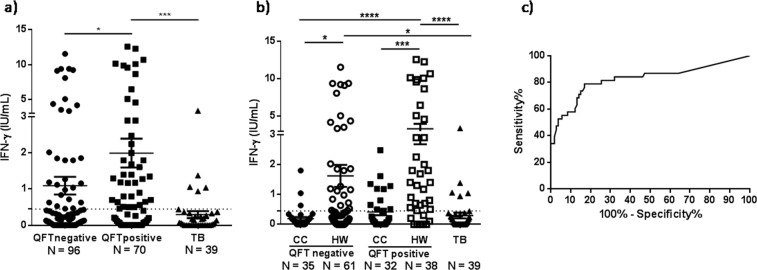
Production of IFN-γ against Rv2626c in whole blood from individuals highly exposed to *Mycobacterium tuberculosis*. (**a**,**b**) Whole blood was incubated with Rv2626c antigen (2.5 μg/mL) for 24 h. Afterwards, plasma samples were collected and IFN-γ production was evaluated by ELISA. The IFN-γ levels are expressed as IU/mL (IU_IFN-γ_/mL = IU_IFN-γ_/mL measured in Rv2626c tube – IU_IFN-γ_/mL measured in Negative control tube). Mean ± SEM for each group of subjects is shown. Each symbol represents an individual. *P < 0.05, ***P < 0.001, ****P < 0.0001. Kruskal-Wallis test and Dunn’s multiple comparisons post-test for unpaired samples. The horizontal dotted line indicates the Rv2626c IGRA cut-off (0.45 IU_IFN-γ_/mL). (**c**) ROC (Receiver Operating Characteristic) analysis for the IFN-γ responses to Rv2626c was performed to evaluate its potential use in discriminating LTBI (HW QFT positive) from other *Mtb* exposed individuals (CC QFT negative and positive and TB). AUC = 0.8258; *P* < 0.0001; 95% CI: 0.7348–0.9169.

Therefore, since we previously demonstrated that *Mtb* dormancy antigen Rv2626c differentiates latently infected BCG-vaccinated individuals^13^, we then assayed the correlation between QFT or TST results and the data obtained with a homemade Rv2626c IGRA. Briefly, we stimulated whole blood from 205 individuals (including CC, HW and TB patients) with *Mtb* Rv2626c antigen and 24 h after, IFN-γ production was determined by a commercial ELISA kit (BioLegend, USA). QFT assay was also performed in the same subjects. To analyze the correlation between Rv2626c IGRA and TST, we compared the results obtained with 118 individuals tested for both assays. Figure 2a shows that the established cut-off threshold for the Rv2626c IGRA was 0.45 IU_IFN-γ_/mL (Sensitivity = 78.95%; Specificity = 83.02%) as defined by a ROC analysis (Fig. 3c), whereas the QFT cut-off was 0.35 IU_IFN-γ_/mL according to the manufacturer. The TST cut-off was 10 mm according to local guidelines (Fig. 2b). Then, we first analyzed the correlation between QTF and Rv2626c IGRA results. Our results indicated that 34% of the studied people displayed negative results for both tests, and 21% of them exhibited double positive results (Percent Concordant = 55%). Moreover, by analyzing the data using the Cohen´s Kappa statistic coefficient, a slight agreement was detected (*kappa* = 0.106; SE of *kappa* = 0.066). Next, we examined the correlation between the TST results and the Rv2626c IGRA data. We observed that 45% of the subjects analyzed presented negative results for both tests, and 16% showed double positive results (Percent Concordant = 61%). Moreover, the analyses using the Cohen´s Kappa statistic coefficient displayed a slight agreement (*kappa* = 0.152; SE of *kappa* = 0.092). Therefore, considering the weak concordance detected by analyzing the results of the available assays and our homemade IGRA, we hypothesized the existence of different responses to Rv2626c antigen from individuals comprised within each group (CC, HW y TB). Since our homemade Rv2626c assay measures IFN-γ by ELISA like QFT kit does, we next classified CC and HW according to QFT.

### **Analysis of IFN-γ responses to Rv2626c latency antigen in people highly exposed to*M. tuberculosis***

We have previously demonstrated that the dormancy antigen Rv2626c discriminates LTBI from healthy people and TB patients^13^. Briefly, subjects with positive QFT and TST results and no clinical or radiological evidence of active TB were classified as LTBI, without considering the exposure period to *Mtb*. In the present work, we confirmed those previous findings showing that non TB QFT positive individuals produced significant higher levels of IFN-γ against Rv2626c than QFT negative subjects and TB patients (Fig. 3a). To further investigate the ability of this *Mtb* dormancy antigen to differentiate latently infected people, in the present work we considered the exposure time of each subject to the pathogen. Interestingly, we observed significantly different IFN-γ responses to Rv2626c between QFT negative and QFT positive individuals. Remarkably, we could establish that those differences were related to the exposure period of the individual to *Mtb* (Fig. 3b). In fact, 69% of CC QFT positive individuals (subjects commonly identified as LTBI) did not secrete IFN-γ in response to the specific *Mtb* latency antigen Rv2626c (Fig. 3b). On the other hand, 43% of HW QFT negative subjects (individuals that were not infected with *Mtb* according to QFT) responded to Rv2626c, as indicated by the levels of IFN-γ produced against the antigen (Fig. 3b). Furthermore, 79% of HW QFT positive subjects secreted elevated levels of IFN-γ when their blood´s cells were cultured with the latency antigen Rv2626c (Fig. 3b). Interestingly, and in contrast with these findings, stimulation of blood from TB patients and HW QFT positive individuals with HspX, another *Mtb* specific antigen, induced a significant IFN-γ production from both groups of subjects, but no differences could be detected between active and latent infected groups (data not shown). Therefore, our findings reflect that once *Mtb* enters the host, the pathogen requires a period of time to establish the dormancy state and secrete antigens like Rv2626c.

In view of our results, and to evaluate the potential use of Rv2626c to discriminate individuals with established latency from non - LTBI subjects, we performed a ROC analysis for IFN-γ responses. From those studies, significant results were obtained (AUC = 0.8258; *P* < 0.0001; 95% CI: 0.7348–0.9169), demonstrating that Rv2626c allows differentiating LTBI individuals from other subjects exposed to *Mtb*, either infected or not infected (cut-off = 0.45 IU_IFN-γ_/mL; Sensitivity = 78.95%; Specificity = 83.02%).

As mentioned above an important number of HW QFT negative subjects (43%) displayed IFN-γ responses to Rv2626c antigen (Fig. 3b), indicating that many individuals with established *Mtb* latency infection (LTBI) could not be differentiated by QFT.

### **IgG responses to Rv262c antigen in close contacts, healthcare workers and TB patients**

**Figure 4:**
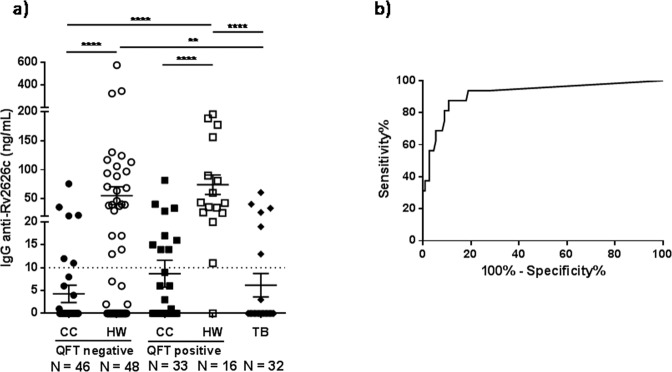
Antibody responses to Rv2626c antigen in plasma from individuals highly exposed to *Mycobacterium tuberculosis*. (**a)** Samples from CC, HW and TB patients were collected and total IgG responses were measured by ELISA. Each symbol represents the levels of anti-Rv2626c IgG per individual (ng/mL). Mean ± SEM for each group of subjects is shown. **P < 0.01, ****P < 0.0001. *P*-values were calculated by Kruskal-Wallis test and Dunn’s multiple comparisons post-test for unpaired samples. The horizontal dotted line indicates the IgG ELISA cut-off (10 ng/mL). **(b)** ROC (Receiver Operating Characteristic) analysis for IgG responses to Rv2626c was performed to evaluate its potential use in discriminating LTBI (HW QFT positive) from other *Mtb* exposed individuals *(*CC QFT positive and QFT negative and TB). AUC = 0.9186; *P* < 0.0001; 95% CI: 0,8358–1,001.

We also evaluated the levels of IgG against Rv2626c antigen in plasma from individuals included in the study groups. Thus, using a homemade ELISA, we measured the levels of IgG anti-Rv2626c in plasma from CC, HW and TB patients. Interestingly, IgG anti-Rv2626c results showed the same trend than IFN-γ data. Accordingly, CC QFT negative, CC QFT positive individuals and TB patients displayed the lowest levels of IgG against Rv2626c. In contrast, HW QFT positive subjects presented the highest levels of IgG anti-Rv2626c (Fig. 4a). Moreover, HW QFT negative displayed significant higher levels of IgG against Rv2626c as compared to patients with active *Mtb* infection or CC QFT negative individuals (Fig. 4a). Actually, 54% of HW QFT negative people presented plasma IgG response to Rv2626c antigen (Fig. 4a), in accordance with our IFN-γ results (Fig. 3b). Furthermore, the ROC analysis for IgG responses to Rv2626c in plasma reinforced the potential of Rv2626c antigen for discriminating individuals with established *Mtb* latency infection from other non-LTBI individuals (Fig. 4b, AUC = 0.9186; *P* < 0.0001; 95% CI: 0.8358-1.001; cut-off = 10 ng_IgG_/mL; Sensitivity = 93.75%; Specificity = 81.08%).

Together, our present findings indicate that QFT fails to identify a significant number of LTBI individuals. Furthermore, we demonstrate that many QFT positive individuals do not respond to a specific dormancy *Mtb* antigen, impairing the confirmation of latent infection. In conclusion, either by using the Rv2626c IGRA or by measuring anti-Rv2626c IgG antibodies we were able to diagnose established *Mtb* latent infection.

## Discussion

Twenty-eight years ago, the WHO issued a press release announcing that TB was a global emergency^14^. Although progress has been made since then, no country has eliminated TB and almost 2 billion persons are now latently infected with *Mtb*^1^. In order to achieve disease control, a reliable diagnostic method for latent infection is required. Currently, both TST and IGRAs are used for the diagnosis of *Mtb* infection. However, these tests do not discriminate active from latent infection and no gold standard for LTBI diagnosis is available^15^. Thus, improved assays to diagnose latent and active *Mtb* infection are immediately required. In line with this, we previously demonstrated that whole blood stimulation with Rv2626c allowed to differentiate QFT positive individuals from TB patients and healthy donors^13^. Recently, Singh *et al*. showed that peripheral blood mononuclear cell (PBMC) stimulation with Rv2626c induced 2% of CD4^+^IFN-γ^+^ T lymphocytes in TB patients but no response in healthy donors. However, the authors provided no information about TST and/or QFT results for the study subjects^16^. Besides, Prabhavathi *et al*. reported that cell stimulation with Rv2626c produced significant levels of IFN-γ against Rv2626c in QFT positive healthy household contacts whereas TB patients did not respond to this antigen^17^. In contrast to our present study, Prabhavathi *et al*.´s work did not include QFT negative household contacts and QFT positive/negative HW individuals. Furthermore, QFT positive healthy household contacts comprised individuals exposed to *Mtb* for at least three months^17^. On the other hand, in a proof-of-principle study, Goletti *et al*. reported that cell stimulation with Rv2626c did not induce IFN-γ responses from subjects with recent or remote *Mtb* infection^18^. In contrast to our present data, Goletti *et al*.´s work included a limited number of individuals with only 4% of Latin American subjects^18^.

In the present work, we extended our previous studies to further investigate whether Rv2626c antigen might be used to identify subjects with established latent tuberculosis infection. Both TST positive and IGRA positive results might refer to an heterogeneous group of individuals: subjects who have subclinical disease; people with persistent, lifelong infection and active immune control (“true” latent infection); those who temporarily contain *Mtb* infection but then develop active disease (caused by immunosuppression for example); persons who progress to primary active disease and individuals that could effectively clear the pathogen with development of detectable T cell mediated adaptive response (uninfected persons)^8^. Therefore, the existence of this wide spectrum of TST positive/IGRA positive individuals clearly reflects that QFT positive results do not indicate latent infection, especially in tuberculosis endemic countries. Therefore, besides ESAT-6 and CFP-10 QFT antigens, it urges to find new *Mtb* antigens, which might complement or improve current assays to diagnose both latent and active infections.

LTBI is related to a dormancy state of *Mtb* and various antigens up-regulated during latency have been proposed as potential diagnostic biomarkers. Accordingly, we have reported the immune-stimulatory role of Rv2626c, a highly expressed protein during hypoxic conditions. Although Rv2626c can be identified in culture filtrates and lysates of *Mtb*^19^, its role remains to be elucidated. Variable results using Rv2626c latency antigen were reported by others^9,16–18,20^, although the disparities could be related to differences in stimulation schemes, antigen concentrations, ethnic backgrounds and BCG vaccination history^13^.

In our present study 22% of the individuals assessed had discordant results between QFT and TST results. Moreover, a subpopulation of QFT negative individuals secreted significant IFN-γ levels against Rv2626c (90% HW exposed to *Mtb* for more than two years). On the other side, we observed that 43% of QFT positive non-TB subjects did not respond to Rv2626c stimulation (73% CC exposed to *Mtb* for less than three months).

Although the importance of humoral immunity in TB has been commonly neglected^21^, recent evidence indicated that B lymphocytes can modulate the host response to various intracellular pathogens including *Mycobacterium* spp^22^. In this regard, Coppola *et al*. reported that IgG levels against Rv1733c *Mtb* antigen correlated with control of TB infection and progression, while other *Mtb* antigen-specific antibodies correlated with TB disease activity and bacillary loads^23^. Besides, it has been proposed that, since *Mtb* can change its physiological state during different stages of infection, showing a differential expression of immunogenic antigens, latent or active TB might be identified by specific antibody profiles^24^. Accordingly, in order to differentiate active TB from LTBI or controls, de Araujo *et al*. analyzed the specific IgG subclasses elicited in response to a set of mycobacterial antigens. Based on their findings, the authors suggested that antibodies subclasses would be differentially modulated during *Mtb* infection^24^. In addition, the poor discriminatory power of current serodiagnostic tests in regions of endemicity has been reported^25^. This might be explained by a significant antibody response against some *Mtb* antigens in latently infected contacts, as well as TB patients, particularly in areas with high levels of exposure to *Mtb*^25,26^. Previously, two different groups measured high levels of circulating antibodies against Rv2626c in TB patients^27,28^. However, none of those studies included a significant number of LTBI QFT and/or TST positive subjects to support their findings. In the present work, we detected the highest levels of plasma anti-Rv2626c IgG in subjects with established latent infection, whereas the lowest amounts of these antibodies were measured in recently infected CC and TB patients (Fig. 4a). Interestingly, the pattern of plasma anti-Rv2626c IgG detected in the study groups was consistent with our data regarding IFN-γ secretion to Rv2626c.

Pollock *et al*. previously reported that QFT failed to detect LTBI among HW^29^. Thus, the authors suggested that QFT negative results should be interpreted with quite caution. Furthermore, Park *et al*. demonstrated important fluctuations in the IGRAs results from high *Mtb* exposed HW tested during one year^30^. Those oscillations were related either to a poor reproducibility of the QFT assay or to a periodic secretion of antigens from *Mtb*. Our findings indicate that QFT fails to identify a significant number of *Mtb* infected individuals that display specific IFN-γ and IgG responses against Rv2626c. Together, our data demonstrate that subjects with established latent *Mtb* infection might be discriminated from individuals recently exposed to the pathogen by analyzing their immune responses to Rv2626c *Mtb* antigen. Therefore, considering previous published data and our present results we hypothesize that the use of a dormancy antigen such as Rv2626c could be an advantageous tool for the improvement of the diagnosis of established LTBI. An assay including this latency antigen would reduce false QFT negative results for individuals that have been long exposed to *Mtb*, allowing proving an established latency state in infected people.

## **Methods**

### **Study subjects**

For this study, individuals exposed to *Mycobacterium tuberculosis* (*Mtb*) during different periods or subjects infected with the bacteria were recruited. Therefore, the following groups of individuals were included: 1) Close contacts (CC): subjects who had lived or worked for less than three months, during 6 or more hours each day, with recently diagnosed pulmonary tuberculosis patients. 2) Healthcare workers (HW): physicians and nurses who had worked at least two years at Hospital areas where tuberculosis patients were confined. 3) Tuberculosis patients (TB): subjects diagnosed with active disease as evaluated at Dr. F. Muñiz or P. Piñero Hospitals (Buenos Aires, Argentina). Diagnosis was established based on clinical and radiological data together with culture-confirmation. None of the patients included in this study had displayed multidrug-resistant (MDR) or extremely drug resistant (XDR) tuberculosis. The participating tuberculosis patients had received less than one week of anti-TB therapy (rifampicin, pyrazinamide, ethambutol and isoniazid). Information regarding demographic data was obtained at the time of recruitment through a structured questionnaire. All participants provided written informed consent for sample collection and subsequent analysis. The protocols conducted through the present work were approved by the Ethical Committees of the Dr. F. Muñiz and the P. Piñero Hospitals. All methods were carried out in accordance with relevant guidelines and regulations.

### Study inclusion and exclusion criteria

Inclusion criteria: a) adult (over 18 years old) men and women with active pulmonary TB and b) healthy volunteers with high level of exposure to *Mtb* (CC of TB patients and HW from Public Hospitals). All enrolled subjects in this study had been BCG-vaccinated. *Mtb* positive cultures were obtained for all TB patients included in this study.

Exclusion criteria: a) HIV positive infection or positive serology for other viral (e.g., hepatitis A, B or C), or bacterial infections (e.g., leprosy, syphilis, etc); b) patients with diabetes, cancer, autoimmune diseases or other conditions that may affect the immune system of the individual; c) pregnant women and d) children. Among the population of TB patients, we excluded: a) patients with multidrug-resistant tuberculosis (MDR) infection or extremely drug resistant (XDR) tuberculosis and b) patients with more than seven consecutive days of anti-TB treatment.

### **Tuberculin skin test**

TST was administered by the medical staff according to the Mantoux procedure. Briefly, two tuberculin units (0.1 ml) of purified protein derivative (PPD, Instituto Nacional de Mirobiología Dr. Malbrán, Buenos Aires, Argentina) were injected by intradermal route into the inner surface of the forearm. The skin induration was measured (in mm) after 48–72 h by trained personnel. A positive TST was defined as an induration size ≥ 10 mm, according to National Guidelines^31^. In CC individuals, TST was performed immediately (less than 7 days) after tuberculosis diagnosis of the index case. Furthermore, in TB patients, TST was performed before the start of antibiotic treatment. In the case of HW, the TST assay was performed immediately after blood extraction for the QFT test.

### **QuantiFERON-TB Gold Plus (QFT) Assay**

QFT (Qiagen, Germany, USA) test and data interpretation were performed according to the manufacturer´s instructions. QFT results were recorded positive if a single antigen tube (either TB1 or TB2) was ≥ 0.35 IU/mL as compared to the negative control (“Nil” tube). In CC subjects, QFT assay was performed immediately (less than 7 days) after tuberculosis diagnosis of the index case. Furthermore, in TB patients, QFT was performed before the start of antibiotic treatment.

### **Rv2626c antigen**

Recombinant antigen Rv2626c was produced as previously described^13^. Briefly, the protein was expressed in *Escherichia coli* BL21 (DE3) pLysS and purified using a nickel nitrilotriacetic system (Ni-NTA Agarose, Qiagen, USA), following the manufacturer´s instructions. After purity control, the protein was concentrated using Amicon (Merck Millipore, USA) commercial columns and then Detoxi-Gel Endotoxin Removing Resin (Pierce, USA) were used to remove possible endotoxin traces.

### **Rv2626c interferon gamma release assay (Rv2626c IGRA)**

A new IGRA using Rv2626c as antigen was developed for this work. The same blood sample obtained from each participant was used to perform Rv2626c IGRA and QFT. Rv2626c IGRA consists of the following two steps:

#### Whole blood stimulation

Heparinized whole blood (500 µL) was seeded in 5 mL sterile round-bottom polystyrene tubes with caps (Falcon, USA) and Rv2626c antigen (2.5 µg/mL), phytohaemagglutinin (PHA-P, 5 µg/mL, positive control, Sigma-Aldrich, USA) or HBSS buffer (negative control) were added and incubated for 16–24 h at 37 °C. Afterwards, plasmas were recovered to determine IFN-γ production by ELISA.

#### *IFN-γ determination*

The levels of IFN-γ were evaluated using a commercial ELISA kit (Human IFN-γ ELISA MAX Standard Kit, BioLegend, USA) following the manufacturer´s instructions. Data are shown as international units of IFN-γ per milliliter (IU_IFN-γ_/mL) after subtracting the negative control value.

### ELISA to determine plasma anti-Rv2626c IgG

High binding ELISA plates were coated with recombinant Rv2626c at 2.5 μg/mL or different dilutions of a human IgG antibody standard (calibration curve; Vigam Liquid Inmunoglobulina Humana Normal). Plates were blocked with 2% skim milk in saline solution. Samples (plasma obtained from heparinized non-stimulated whole blood) were diluted 1:100 and run in duplicate. Then, a secondary antibody (biotin mouse anti-human IgG - BD Pharmingen, USA) was added. Plates were consequently incubated for 1 h at 37 °C. High sensitivity streptavidin - horseradish peroxidase enzyme (Thermo Fisher Scientific, USA) was incorporated and 3, 3′5, 5′-tetramethylbenzidine (TMB) (Sigma-Aldrich, USA) substrate was added. Finally, sulfuric acid was incorporated to stop the enzyme reaction and plates were read at 450 nm. The results are expressed as ng/ml of IgG.

### **Statistical analysis**

In all the graphs, each symbol represents an individual. Moreover, data were expressed as mean ± standard error of the mean (SEM). The Kruskal – Wallis (K-W) followed by Dunn’s multiple comparisons tests for unpaired and non-parametric samples were used to analyze differences between groups. Monte Carlo simulations for the chosen K-W methods resulted in a minimum group size of 15 for statistical power > 0.8. Briefly, a K-W Rank Sum method was performed on simulated data and the resulting *P*-value was recorded. This process was repeated 10.000 times at each group size (N) evaluated, from 2 to 50. And we found that at N = 15, the proportion of *P*-values < 0.05 was greater than 0.8 (P = 0.81). Therefore, the results from K-W tests performed on data with similar distribution to that recorded by the experimenters and with group sizes larger than 15 have more than 80% probability of rejecting the null hypothesis on the K-W test.

Receiver operating characteristic (ROC) curve analysis was performed to analyze the predictive value of IFN-γ response to Rv2626c, or anti-Rv2626c IgG response, calculating the area under the curve (AUC) and the 95% confidence interval (CI). Comparison between the diagnostic methods (QFT / TST, QFT/Rv2626c IGRA and TST/Rv2626c IGRA) was made using the Cohen’s *Kappa* Statistic test. Analyses were performed using GraphPad Prism 7.00 software. *P* < 0.05 was considered statistically significant.
